# Doppel as an early‐stage biomarker promoting EMT and dissemination in ovarian cancers

**DOI:** 10.1002/ijc.70268

**Published:** 2025-11-29

**Authors:** Zulfikar Azam, Xiaojun Zhang, Riajul Wahab, Md Mahedi Hasan, Shivaniben Dhadhal, Bowon Kang, Md Mynul Hassan, Mazharul Karim, Jeong Uk Choi, Muhit Rana, Jian‐Ying Zhang, Sourav Roy, Youngro Byun, In‐San Kim, Jae Yun Song, Eugene P. Toy, Sireesha Y. Reddy, Farzana Alam, Taslim A. Al‐Hilal

**Affiliations:** ^1^ Department of Molecular Pharmaceutics University of Utah Salt Lake City Utah USA; ^2^ Department of Pharmaceutical Sciences, School of Pharmacy University of Texas at El Paso El Paso Texas USA; ^3^ Department of Biological Sciences, College of Sciences University of Texas at El Paso El Paso Texas USA; ^4^ Department of Biomedical Engineering University of Utah Salt Lake City Utah USA; ^5^ Deptartment of OB/GYN College of Medicine, Korea University Seoul Republic of Korea; ^6^ College of Pharmacy Kyung Hee University Seoul Republic of Korea; ^7^ Giner Inc. Newton Massachusetts USA; ^8^ Research Institute of Pharmaceutical Sciences College of Pharmacy, Seoul National University Seoul Republic of Korea; ^9^ KU‐KIST Graduate School of Converging Science and Technology Korea University Seoul Republic of Korea; ^10^ Biomedical Research Institute Korea Institute of Science and Technology (KIST) Seoul Republic of Korea; ^11^ Division of Gynecology Oncology Texas Tech University Health Sciences Center El Paso Texas USA; ^12^ Department of Pharmacology and Toxicology University of Utah Salt Lake City Utah USA

**Keywords:** biomarker, circulating tumor cells, Doppel, EMT, organoids, ovarian cancer

## Abstract

Detecting ovarian cancer (OC) early using existing biomarkers, for example, cancer antigen 125 (CA125), is challenging due to its ubiquitous expression in many tissues. Doppel, a prion‐like protein, expresses in the male reproductive organ but is absent in female reproductive systems and healthy tissues, but plays an important role in neo‐angiogenesis. Here, we have shown two platforms, soluble Doppel in sera/ascites and Doppel expressed in circulating tumor cells (^Dpl+^CTC) in the whole blood, to detect subsets of epithelial OC (EOC). Increased levels of Doppel in the sera of OC patients, in three different cohorts, confirm Doppel as an OC‐specific biomarker. Serum Doppel levels can distinguish OC with high sensitivity and specificity (sensitivity = 0.91 and specificity = 0.89) and can also detect early‐stage HGSOCs (FIGO stages I and II) from non‐cancerous conditions with high sensitivity and specificity (sensitivity = 0.94 and specificity = 0.83). Moreover, significantly higher Doppel expression is observed in all EOC subtypes except clear cell OC. Stratifying the EOCs based on Doppel levels, we categorized them into Doppel‐high (Dpl^hi^) and Doppel‐low (Dpl^low^) groups. Using ascites‐derived organoids, made from Dpl^hi^ and Dpl^low^ patients, we identify that Doppel induces epithelial–mesenchymal transition (EMT). Doppel levels in the sera/ascites correlate with the changes in ^Dpl+^CTC number in whole blood, highlighting the association of Doppel‐induced EMT with CTC dissemination in the circulation. Thus, Doppel‐based detection of EOC subtypes could be a promising platform as a clinical biomarker and link the Doppel axis with OC dissemination.

## Graphical abstract

What's new?

Existing biomarkers for ovarian cancer (OC) lack the sensitivity and specificity needed to consistently distinguish the malignancy from non‐ovarian influences and other cancers. Here, the authors explored the ability of the prion‐like protein Doppel, normally found in the male testes, to detect OC. Analyses show that Doppel is specifically expressed in OC tissues, being detectable in serum and ascites. In the latter, it further promotes epithelial–mesenchymal transition events, and in serum, its levels correlate with Doppel+ circulating tumor cell concentrations, suggesting that it facilitates OC dissemination. The findings highlight Doppel's potential as a diagnostic marker and driver of OC progression.

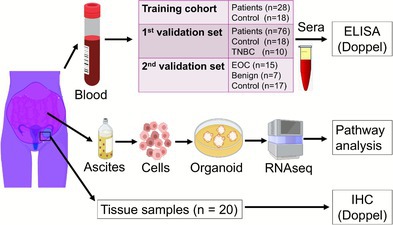

**Abbreviations**
AUC: area under curve
CA125: cancer antigen 125
CK7: cytokeratin 7
CTC: circulating tumor cells
Dpl + CTC: Doppel expressed circulating tumor cells
Dpl^hi^
: Doppel‐high
Dpl^low^
: Doppel‐low
ELISA: enzyme‐linked immunosorbent assay
EMT: epithelial–mesenchymal transition
EOC: epithelial OC
EpCAM: epithelial cell adhesion molecule
FDA: Food and Drug Administration
GTEX: genotype‐tissue expression
HE4: epididymis protein 4
HGSOC: high‐grade serous ovarian carcinoma
LLOD: lower limit of detection
OC: ovarian cancer
OS: overall survival
PAX8: paired box 8
PRND: Doppel
RFS: regression free survival
TCGA: The Cancer Genomics Atlas
TNBC: triple negative breast cancer
TVUS: transvaginal ultrasound
VEGF: vascular endothelial growth factor
VEGFR2: vascular endothelial growth factor receptor 2

## 1 INTRODUCTION

The latent symptoms and subtle onset of ovarian cancer (OC) pose a significant challenge in identifying the disease at its earlier stages.^1^ Frequent metastasis and failure to detect earlier exacerbate the OC progression and contribute greatly to OC patients' overall survival, which stands at <20%, 5 years survival from disease diagnosis for stage III and IV OC patients.^2^ The high morbidity and mortality of OC patients underscore the importance of identifying new biomarkers which can effectively detect and stratify the course of OC cancer fate. Beyond the detection of OC cancer early, diagnostic methods are also needed to track disease progression, treatment response and treatment resistance. The cancer antigen 125 (CA‐125) and human epididymis protein 4 (HE4), two FDA approved serum glycoprotein biomarkers, are widely used to detect patients with transvaginal ultrasound (TVUS). The utility of CA‐125 as a reliable biomarker for OC diagnosis is less certain than expected. As a glycoprotein CA‐125 is widely expressed in normal tissue types along with non‐cancerous conditions in women, making it less specific for OC diagnosis.^3^ The sensitivity of CA‐125 is not sufficient to detect epithelial ovarian cancer (EOC) with one in five found significantly lower levels of CA‐125.^4^ HE4's ability to distinguish EOC from benign masses is superior to CA‐125. But the level of HE4 is increased by multiple non‐ovarian influences and various malignancies, predominantly those originating from the reproductive system but also encompassing respiratory cancers.^5^, ^6^ Thus, the discovery of a potential selective biomarker that is absent in the sera or reproductive organs of healthy females but present in ovarian cancer patients is immensely needed.

Doppel, encoded by the *PRND* gene, is a 179‐amino‐acid polypeptide with domains similar to those of cellular prions (PrP), sharing 25% structural homology with PrP.^7^ Based on amino acid pairing, human Doppel has 79% similarity with mouse Doppel.^7^ Doppel was first identified in 1999 and later discovered to be expressed in human adult testis and therefore is believed to play a role in male fertility.^8^, ^9^ Recent quantitative transcriptomic analysis (RNA‐seq) of the human genome confirmed the testis‐specific expression of Doppel.^10^ Although the brain endothelium of newborn mice expresses Doppel transiently, the endothelium of the adult mouse brain expresses no Doppel.^11^ Doppel knock‐out mice, created from full immunogenic mice, exhibit no developmental defects except sterility in males, suggesting that Doppel may have no role in the development and physiology of female reproductive systems.^12^ We and others reported that Doppel is a highly specific tumor endothelium marker.^13^, ^14^ We showed that both human and mouse tumors express Doppel in their vasculatures; inhibition of Doppel depletes cell‐surface vascular‐endothelial growth factor receptor 2 (VEGFR2), and thus abrogates vascular‐endothelial growth factor (VEGF) binding with the receptor.^14^

**FIGURE 1:**
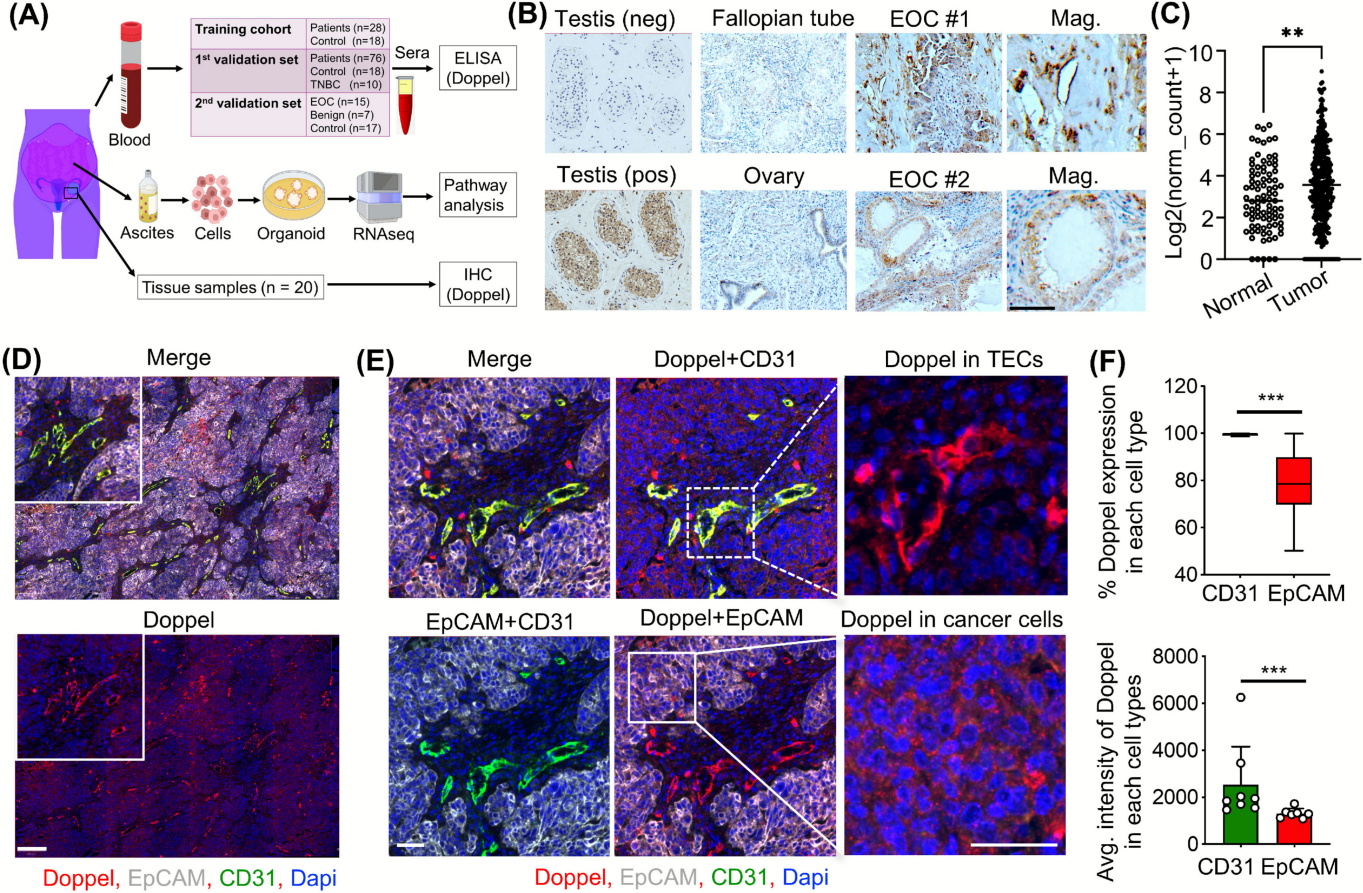
Doppel is present in endothelial and epithelial cell populations across ovarian cancer tissues. (A) Schematic of overall study design. (B) Representative IHC images of tissue samples collected from testis, normal fallopian tube and ovary and OC tissue samples. Testis tissue samples are used for validation purposes. Scale bar: 50 μm. EOC#1 image is reused in Figure S2 as ID#495 and Ovary image is reused in Figure S1C as Ovary. (C) TCGA TARGET GTEx ovary RNA sequencing data comparison between serous OC patients and healthy tissue samples. Each dot indicates an individual subject. (D and E) Representative images of multiplex IF showing the expression of Doppel in endothelial and epithelial cells in OC tissue samples. Scale bars: 200 and 50 μm. (F) The percentage of Doppel co‐expression in endothelial and epithelial cell populations is shown in the top bar chart, while the average intensity of Doppel co‐expression in these populations is displayed in the bottom bar chart. **P < .01, ****P < .0001.

In this study, we seize the opportunity of this absent expression of Doppel in the female reproductive organ as well as in any healthy tissues other than the male testis, to establish Doppel as an OC‐specific diagnostic biomarker by utilizing clinical specimens across three cohorts (Figure 1A). We elaborated our findings to correlate Doppel's role in the epithelial to mesenchymal transition (EMT) of OC by utilizing the power of ascites‐derived organoids and identifying Doppel‐expressed circulating tumor cells (^Dpl+^CTCs) in retrospective patient samples.

## 2 MATERIALS AND METHODS

### 2.1 Collection of clinical samples

We collected clinical samples from three different cohorts. For the training cohort, sera collected from 28 ovarian cancer patients and 18 healthy female subjects were analyzed from an archived cohort.^15^ For the 1st validation cohort, sera collected from 76 ovarian cancer patients, 10 triple‐negative breast cancer (TNBC) patients, and 18 healthy females were analyzed. Serum samples were collected at the Korea University Anam Hospital from 2006 to 2020 from patients who underwent conservative, comprehensive or staging surgery for ovarian cancer. Preoperative imaging procedures (ultrasonography and computed tomography) were used to identify the presence of ovarian cancer with stages from I to IV. In the 2nd validation cohort, 15 ovarian cancer and 7 benign cancer patients were recruited, and specimens were collected during surgery at Texas Tech University Health Sciences Center El Paso under Institutional Review Board‐approved protocols (IRB#: E21144) between October 2021 and December 2023. The disease stages of recruited patients were clearly defined in the IRB protocol, based on the 2018 International Federation of Gynecology and Obstetrics classification system. *Patient inclusion criteria*: patients between 35 and 85 years of age with no severe autoimmune disease or previous history of cancer were included. Ovarian cancer cohorts comprise benign, borderline (BL) cases, type I cases and type II cases, and stages I to IV. Type I cases and type II cases were clearly defined in the IRB protocol. *Patient exclusion criteria*: patients with a previous history of familial ovarian cancer and ages below 35 years of age were excluded from the analysis. Patient‐matched control sera were collected from 17 healthy females. *Control inclusion criteria*: 35–85 years of age control subjects with no prior history of seizure disorder, head trauma (within <1 year), cancer, CNS tumor, hepatic cirrhosis, connective tissue disease (i.e., lupus, rheumatoid arthritis, etc.), congenital heart disease were considered healthy controls. *Control exclusion criteria*: the following exclusion criteria were maintained while recruiting control subjects—women under the age of 35, planned surgical intervention, decline phlebotomy, self‐reported previous bilateral oophorectomy, subjects diagnosed with cancer. Blood and ascites were drawn and drained respectively by an interventional radiologist and transferred for further processing in appropriate containers. The demographics of these patients are summarized in Table S1.

### 2.2 Cell line

SK‐OV‐3 (RRID: CVCL_0532) cell line was purchased from ATCC and cultured according to ATCC protocols. All experiments were performed with mycoplasma‐free cells. The human cell line SK‐OV‐3 has been authenticated using STR profiling within the last 3 years.

### 2.3 Enzyme‐linked immunosorbent assay (ELISA)

Serum and ascites Doppel level was measured using Human Prion‐like protein Doppel ELISA Kit, 96‐Strip‐Wells (MBS76513, MyBioSource, CA) according to the manufacturer's instructions. Briefly, we aliquoted 100 μL of standards and 100 μL of diluted samples into a pre‐coated plate, incubated the sealed plate at 37°C for 90 min, and then washed twice with wash buffer. Exactly 100 μL biotin‐labeled antibody solution, 100 μL HRP‐streptavidin conjugate and 90 μL TMB substrate were added sequentially to each well. Between each step, incubation and washing steps were performed following protocols. At the end of the experiment, we stopped the reaction with 50 μL stop solution and immediately measured the absorbance at 450 nm. The following formula was used to measure the sample concentration: (the relative OD 450) = (the OD 450 of each well) − (the OD 450 of the blank well). A standard curve was made by plotting the relative OD 450 of each standard solution vs. the respective concentration of the standard solution. To fit the concentration of Doppel within standard, we made 2 dilutions of each sample (1:20 and 1:40) with triplicates. The final concentration was measured by multiplying the dilution factor by the concentration from interpolation and calculated the average result considering both dilution and triplicate samples of each. For each sample, we conducted four replicates and calculated the concentration as the average of these measurements.

### 2.4 Bioinformatics analysis

Human ovarian cancer Doppel gene‐expression TCGA data (*n* = 419) and normal Doppel gene‐expression ovary data (*n* = 88) were downloaded from Xena Browser (https://xenabrowser.net/) and Doppel expression differences between these two groups were calculated in GraphPad Prism 10. Overall and progression‐free survival associated with Doppel expression in ovarian cancer were performed in Kaplan–Meier Plotter (https://kmplot.com/analysis/), a web‐based survival analysis platform of various cancers. For survival analysis, we chose PRND gene (affymetric ID 222106_at and 223813_at) with percentile survival option and chose all histology to determine the overall and regression‐free survival associated with Doppel expression.

### 2.5 Immunohistochemistry, immunofluorescence, and quantitative PCR

Seven tissue samples were collected from Tech University Health Sciences Center El Paso under Institutional Review Board‐approved protocols (IRB no.: E21144) and 13 tissue samples were purchased from The National Disease Research Interchange (NDRI). Multiplex immunofluorescence, immunohistochemistry and qRT‐PCR were run according to the methods established in our lab previously.^16^ Each experiment is conducted as biological triplicates for qRT‐PCR. The following custom‐made anti‐Doppel monoclonal antibodies were used for immunohistochemistry and immunofluorescence studies: 2B6, 2C10, and 7G3 (AbClon Inc., South Korea). Anti‐Doppel polyclonal antibody (HPA043373) was purchased from Sigma. Other antibodies, such as CD31 (PA5‐16301, Invitrogen), EpCAM (14452S, Cell Signaling), were also used. The following human primer pairs are used: EPCAM_F‐GCCAGTGTACTTCAGTTGGTGC, EPCAM_R‐CCCTTCAGGTTTTGCTCTTCTCC, CLDN1_F‐GTCTTTGACTCCTTGCTGAATCTG, CLDN1_R‐CACCTCATCGTCTTCCAAGCAC, OCLN_F‐ATGGCAAAGTGAATGACAAGCGG, OCLN_R‐CTGTAACGAGGCTGCCTGAAGT, CDH2_F‐CCTCCAGAGTTTACTGCCATGAC, CDH2_R‐GTAGGATCTCCGCCACTGATTC, VIM_F‐AGGCAAAGCAGGAGTCCACTGA, VIM_R‐ATCTGGCGTTCCAGGGACTCAT, ZEB1_F: GGCATACACCTACTCAACTACGG, ZEB1_R‐TGGGCGGTGTAGAATCAGAGTC, TWIST_F‐GCCAGGTACATCGACTTCCTCT, TWIST_R‐TCCATCCTCCAGACCGAGAAGG. The calculation of the total number of Doppel+, CD31+, and EPCAM+ cells in the immunofluorescence data was calculated in QuPath (0.5.1).

Whole tissue sections (*N* = 8) were scanned to calculate single‐cell intensity values, from which the percentage of Doppel‐positive cells was derived. For each tissue, the mean Doppel intensity (±SD) was quantified separately in CD31^+^ and EpCAM^+^ cell populations, and the results were plotted accordingly. The following intensity thresholds were applied to identify positive cells: DAPI – 3500, CD31 – 2000, Doppel – 1000, and EpCAM – 2000. These thresholds were determined based on expression levels observed in positive control testis samples.

### 2.6 Preparation of serum, ascites, and development of organoid model

Freshly collected blood samples in K2EDTA vacutainer tubes were centrifuged for 20 min at 4°C at 1000*g* and the clear supernatant (serum) was collected and stored aliquoted at −80°C for future experiments. Malignant ascites samples were collected fresh from ovarian cancer patients undergoing surgery. The ascitic fluid was centrifuged at 1600*g* for 10 min at 4°C and the supernatant was collected and stored at −80°C for future experiments. To remove the red blood cells (RBC), pelleted cells were resuspended and incubated with diluted RBC lysis buffer (sc‐296258, Santa Cruz, TX) for 10 min at 4°C and centrifuged down at the RBC‐cleared cells. If RBCs were still visible in the pellet, a second round of RBC lysis was performed. The resulting pellet was washed with cold PBS and manual cell counting was performed. The samples were divided into many portions based on cell counting. For organoid culture, freshly pelleted cells were resuspended and cultured following modified protocols adapted from published articles.^17^, ^18^ We used Advanced DMEM/F12 medium supplemented with Glutamax and 1% penicillin–streptomycin as a basal medium and then added other supplements to it. The final concentration of the supplements to prepare 50 mL complete media is as follows: 100 μg/mL Primocin, 1.25 mM *N*‐acetylcysteine, 50 ng/mL R‐spondin 1, 100 ng/mL Noggin, 0.5 μM A83‐01, 10 mM Nicotinamide, 100 nM 17‐β‐Estradiol, 10 μM SB202190, 50 ng/mL EGF, 10 ng/mL FGF10, 50 ng/mL NRG‐1, 10 μM Forskolin, and 500 ng/mL Hydrocortisone. This medium can be stored at 4°C for up to 1 month. Before adding the complete media to the organoid culture, ROCK inhibitor (10 μM Y27632) needs to be added per 1 mL media. This helps to increase the survival efficiency of single cells and the formation of organoids. The complete organoid growth media and Matrigel Reduced Growth Factor Basement Membrane extract (Corning) were mixed at a ratio of 30:70. The ascites single cells were properly mixed with it and seeded in a pre‐warmed 6‐well plate. After polymerization (37°C, 5% CO_2_, 15 min) each well of Matrigel droplets was immersed with 2 mL of complete organoid culture medium. Organoid culture medium was exchanged at an interval of 3 days. After 8 to 13 days, the culture media was removed and Matrigel‐containing organoids were dissociated through several steps of mechanical dissociation and using TrypLE express (37°C up to 5 min). Isolated cells were seeded for the next passage or placed in a cell freezing container (Coolcell, −80°C) in 1 mL of Stem Cell freezing media (ATCC) and bio banked at −140°C the next day.

### 2.7 RNA isolation and bulk RNA sequencing analysis

RNA was extracted from mature organoid using combined TRIzol (15596026, Thermo Fisher Scientific, MA) and RNeasy Mini Kit (74104, Qiagen, MD) following the manufacturer's guidelines. The quality of RNA was determined by Nanodrop absorbance (260/280 ratio ≥ 1.8) and RNA integrity value (≥7) analysis. Bulk RNA‐seq was carried out by Novogene using the Illumina NovaSeq6000 platform. The sequencing coverage and quality statistics for each sample are summarized in Table S3. For each organoid we conducted three biological replicates. Adaptor trimming and quality of raw sequencing files were evaluated using FastQC software (0.12.1), considering metrics such as sequence quality scores, sequence duplication, and adapter content to determine if filtering was needed prior to genome mapping. Clean reads were then aligned to the human GRCh38 reference genome using the HISAT2 (2.2.1) graph‐based read aligner. The aligned reads were assembled into transcripts or genes using StringTie software (2.2.0) considering the Fragments Per Kilobase of transcript sequence per Millions base pairs sequenced (FPKM) approach. Differential gene expression analysis was performed on raw counts with the R statistical package DESeq2 (3.19). Genes exhibiting a Log2 fold change (Log2FC) of ≥1, *P*_adj ≤.05, and count ≥10 were deemed significantly upregulated, while those with a Log2FC ≤1, *P*_adj ≤.05, and count ≥10 were considered significantly downregulated. Gene set enrichment analysis, using hallmark gene set, of significantly dysregulated genes was performed by GSEA (4.3.3) software. The heatmap of the targeted pathway was generated in “R” environment (4.3.2) using ComplexHeatmap (2.18.0) package.

### 2.8 Isolation and identification of circulating tumor cells (CTCs)

The herringbone (HB) was designed using CAD software (AutoDesk AutoCAD) and manufactured using the photolithography method based on the previous report.^19^ A silicon wafer imprinted with the HB chip design served as the primary mold for fabricating Polydimethylsiloxane (PDMS) structures. The PDMS gel was concocted by blending the elastomer base (Part A Sylgard 184) with an elastomer curing agent (Part B Sylgard 184) in a proportion of 10:1 (4019862, Dow, MI). Following plasma treatment, the HB chips and PDMS base were conjoined to yield a complete set of PDMS HB chips. These HB chips were sequentially coated with poly (diallyl dimethylammonium chloride) (522376, Sigma Aldrich, MA) and a biotin‐Alg solution (Bio‐Alg) at concentrations of 2 mg/mL, resulting in layers bearing positive and negative charges. Subsequently, a biotin–avidin solution (PI31000, Molecular Probes, OR) at a concentration of 50 μg/mL was applied to the coated chips and incubated for a minimum of 4 h. Capture antibodies were then introduced to these coated HB chips. Biotin conjugated x‐human anti‐Doppel (A05457‐Biotin‐50 μg, Boster Biological Technology, CA) and biotin conjugated x‐human anti‐EpCAM (51985, Cell Signaling, MA) antibodies were employed as capture antibodies within the HB chips. Post antibody coating, the HB chips were left to incubate overnight to facilitate effective binding between avidin and biotin. These HB chips were then utilized to capture circulating tumor cells (CTCs) from patient blood samples. Whole blood samples were processed within 24 h of collection from patients. These samples were prepared by incorporating 0.4 IU heparin (9041081, Thermo Fisher Scientific, MA) solution and diluting the blood with saline at a 1:1 ratio prior to introducing them to the capture antibody coated HB chips. The prepared blood sample was channeled through the HB chips at a flow rate of 3 μL/min for a duration of 60 min, allowing a total of 200 μL of blood to traverse each HB chip. This procedure ensured minimal obstruction during the blood's passage through the HB chips. For each blood sample, a minimum of five HB chips coated with Doppel and EpCAM were employed. Post‐capture, the chips were rinsed (PBS with 0.1% BSA) at a flow rate of 10 μL/min to remove all non‐captured cells. The captured cells were then fixed (4% formaldehyde) and permeabilized (goat serum and 0.3% Triton X‐100) in preparation for staining. The cells were stained with Dapi (62247, Thermo Fisher Scientific, MA), cytokeratin (1:500, 502086852, Biolegend, CA), CD45 (1:100, 502079010, Biolegend, CA) prior to imaging with an EVOS5000 fluorescence microscope. The CTCs were identified as Dapi+/CK+/CD45−.

### 2.9 Statistical analysis

Where applicable, all experiments were conducted at least three times, and the mean and standard deviation (mean ± SD) were calculated. Statistically significant differences involving two groups were assessed using unpaired two‐tailed Student's *t*‐tests. One‐way Analysis of Variance (ANOVA) was utilized to find statistical differences between more than two groups. Results were considered statistically significant at *P < .05, **P < .01, ***P < .001, and ****P < .0001 for all analyses.

## 3 RESULTS

### 3.1 Doppel expresses in both endothelial and epithelial cells of ovarian cancer tissues

To see Doppel expression in OC tissues, we used three custom‐made antibodies (2B6, 2C10, and 7G3) and one commercially available antibody (HPA, Sigma) to stain Doppel in tissues. The data revealed that 3 out of 4 antibodies specifically detected Doppel; however, 2B6 showed the best results (Figure S1A). 2B6 detected Doppel at an optimized dilution of 1:200 at which testis showed positive staining for Doppel but low to negative staining for healthy fallopian tube or ovarian tissues (Figures S1B,C and 1B). Importantly, Doppel staining was high in OC (12 out of 20) (Figure S2) and testis tissues than in the normal fallopian tube and ovary (3 of each) (Figure S1). Out of 20 tissues, only one tissue was negative for Doppel expression (Figure S2). Using public RNA sequencing data of Genotype‐Tissue Expression (GTEX) normal ovary tissue (*n* = 88) and The Cancer Genome Atlas (TCGA) ovarian primary serous adenocarcinoma (*n* = 419), we observed a strong association of Doppel expression with OC pathobiology (Figure 1C). In line with expression differences, the survival differences between *PRND*‐high and *PRND*‐low serous OC groups showed significant differences both overall and progression‐free survival (Figure S3A,B) clearly indicating Doppel's usefulness as a survival indicator of OC. To determine the cell‐specific expression of Doppel, we next performed the multiplex IF staining of OC tissues (*n* = 8). Despite some variability the data demonstrate that Doppel expresses both in endothelial and tumor cells (Figure 1D,E). However, Doppel expression was significantly higher in tumor endothelial (CD31^+^; ±98%) than in epithelial (EpCAM^+^; ±78%) cells (Figure 1F). The intensity of Doppel in each CD31^+^ cell was also significantly higher than in each EpCAM^+^ cell (Figure 1F). This finding aligned with our previous findings^14^ where we demonstrated that the expression of Doppel was predominantly localized in tumor‐associated endothelial cells in primary tissues. Together, these findings indicate Doppel is prognostic of poor survival and tissue expression of Doppel is abundant in OC. Although lower than tumor endothelial cells, the epithelial cancer cell‐specific Doppel expression may suggest a new function of Doppel in cancer biology.

### 3.2 Doppel is specifically detected in the sera of ovarian cancer patients but not in benign or healthy female's sera

Prion proteins are anchored and processed through the secretory pathway, anchored to the cell membrane via a Glycosylphosphatidylinositol anchor, and can be released into the extracellular space through proteolytic cleavage or via exosomes. For example, prion proteins are cleaved at tyrosine residue Y226 by the enzyme A disintegrin and metalloproteinase domain‐containing protein 10 (ADAM10) at the cell surface, releasing a soluble form into the extracellular space, which can then enter the bloodstream.^20^ After noticing the increased presence of Doppel protein in human ovarian carcinomas, we were intrigued to investigate whether Doppel is likewise discharged from the tumor tissues, as is seen in other secretory prion proteins. We detected Doppel concentration in a training cohort of archived sera samples of healthy and OC samples (Figure S4A). In the training cohort, we detected serum Doppel in ovarian cancer patients (*n* = 28) and healthy control (*n* = 18). Doppel level was found to be significantly higher in ovarian cancer patients than in healthy control (14.24 vs. 1.06 ng/mL) and the receiver operating characteristic (ROC) curve between control and ovarian cancer patients confirmed the performance and applicability of Doppel as a sera marker (Figure S4A,B). The statistics between control and patients in archived training cohort samples showed only two samples among 28 patients below cut‐off value (4.77 ng/mL) (Figure S4C), further strengthening the applicability of Doppel as a sera biomarker.

**FIGURE 2:**
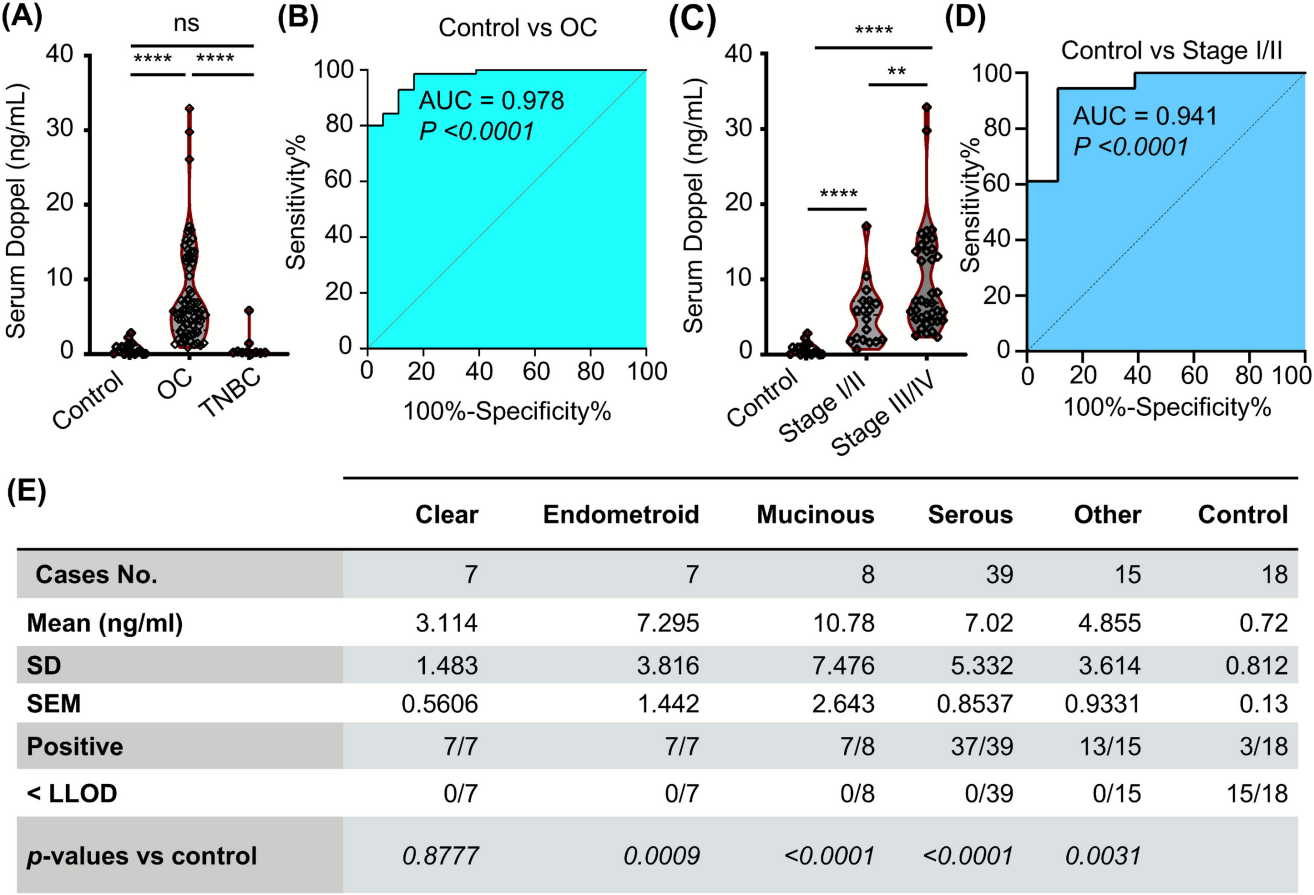
Serum Doppel level in patients with ovarian cancer subtypes. (A) Measurement of serum Doppel level of healthy, OC and TNBC patients by ELISA. (B) The ROC curve of Doppel between control and OC serum samples. (C) Stagewise serum Doppel level in patients with HGSOCs compared with control subjects. (D) The ROC curve of Doppel between control and HGSOC Stage I/II samples. (E) Serum Doppel level based on ovarian cancer histology including epithelial ovarian cancer subtypes (clear cell carcinoma, endometroid, mucinous, and serous). ns = not significant, ****P < .0001; AUC, area under the ROC curve; HGSOC, high grade serous ovarian carcinoma; OC, ovarian cancer; ROC, receiver operating characteristic; TNBC, triple‐negative breast cancer.

To confirm the previous findings, we detected Doppel concentration in the 1st validation set where we used archived sera of 76 OC patients with stage and histopathology information, 10 triple negative breast cancer (TNBC) patients, and 18 healthy females using ELISA. Doppel level was significantly higher in the sera of OC patients than their healthy counterparts (0.720 vs. 6.653 ng/mL, *P* < .0001); 72 out of 76 patients were positive for Doppel expression (cut off 2.67 ng/mL that includes 99% of healthy samples) while only 1 out of 18 cases was positive for healthy females (Figure 2A). Moreover, we did not see the differences between healthy and TNBC samples indicating Doppel specificity for OC diagnosis (Figure 2A). The ROC curve analysis showed that Doppel can differentiate OC patients from normal individuals with an area under the curve (AUC) of 0.978, sensitivity of 0.91, specificity of 0.89 (Figure 2B). Correlation of Doppel level with stages was also observed in HGSOCs patients (Stage I/II 5.28 ± 0.96 ng/mL; *n* = 18 and Stage III/IV 9.64 ± 1.15 ng/mL; *n* = 31 vs. controls 0.72 ± 0.19 ng/mL; *P* < .0001 and *P* < .01 between Stage I/II vs. Stage III/IV) (Figure 2C). The ROC curve analysis also showed that Doppel can differentiate early stage HGSOC patients from healthy individuals with an AUC of 0.941, sensitivity of 0.94, specificity of 0.83 whereas the sensitivity and specificity for late stage HGSOC patients were 1.0 and 0.925, respectively (Figure 2D). Interestingly, serum Doppel level was higher among EOCs of serous adenocarcinoma (7.020 ng/mL; *n* = 39), mucinous (10.780 ng/mL; *n* = 8), and endometrioid (7.295 ng/mL; *n* = 7) than clear cell (3.114 ng/mL; *n* = 7) or other types (4.855 ng/mL; *n* = 15) (Figure 2E). Together, the results from these two archived sera samples showed the potential of Doppel as a serological biomarker for the main subtypes of EOC detection and importantly, for early stage HGSOCs. However, the specificity of Doppel in benign conditions is unknown.

**FIGURE 3:**
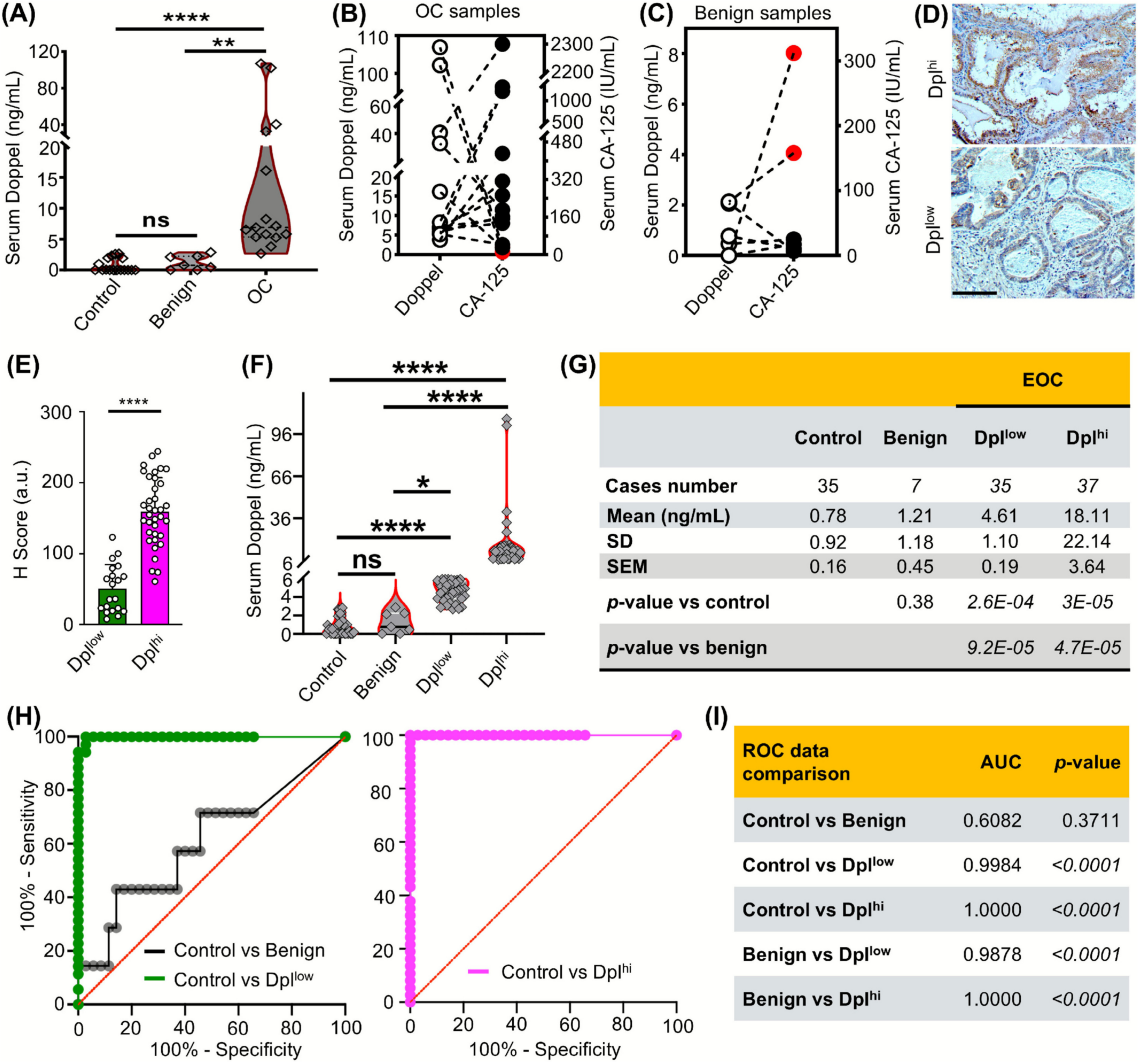
Doppel level distinguishes cancerous from non‐cancerous patients. (A) Comparison of serum Doppel level of OC patients with benign and healthy control samples. (B) Pairwise correlation of serum Doppel with serum CA‐125 level in OC ovarian cancer patients. (C) Pairwise correlation of serum Doppel with serum CA‐125 level in patients with benign tumors. (D) Representative images of tumors from the OC tissue samples with high and low Doppel expression. Scale bar: 50 μm. Dpl^hi^ and Dpl^low^ images in this panel are reused in Figure S2 as ID#494 and ID#491 respectively. (E) H score comparison shown as bar plot between Dpl^low^ and Dpl^hi^ groups. (F) Level of Doppel in healthy, benign and EOC patients that are stratified into Dpl^hi^ and Dpl^low^ groups shown as bar plot. (G) Level of Doppel in healthy, benign and EOC patients that are stratified into Dpl^hi^ and Dpl^low^ groups shown as tabular form. (H) The ROC curve between control vs. benign, control vs. Dpl^low^ and control vs. Dpl^hi^ groups. (I) The AUC and *p*‐value of ROC plots between control vs. benign, control vs. Dpl^low^, control vs. Dpl^hi^, benign vs. Dpl^low^, benign vs. Dpl^hi^ groups. Serum and ascitic Doppel level are measured by ELISA. ns = not significant, *P < .05, **P < .01, ****P < .0001. AUC, area under the ROC curve; EOC, epithelial ovarian cancer; ROC, receiver operating characteristic.

To confirm the specificity of Doppel as an EOC biomarker, we used a 2nd validation cohort which included healthy, EOC and benign patients. We recruited age, race, and ethnicity‐matched 17 control females and treatment‐naive 22 cases (Table S1), out of the 22 cases, 7 are diagnosed as benign and 15 as EOCs. We detected the Doppel level in control, benign and EOC subjects and the results showed EOC subjects' Doppel level significantly higher than control and benign subjects (Figure 3A), whereas no statistical differences are seen between control and benign subjects clearly indicating the Doppel specificity in EOCs. CA‐125, a widely used Food and Drug Administration (FDA) approved marker for OC detection, sometimes fails to detect OCs due to its lower limit of detection (LLOD; <35 IU/mL) and the occurrence of false positive results in benign tumors due to specificity issues.^21^ We compared our Doppel sera findings of EOC and benign subjects with CA‐125; a pairwise comparison revealed that the CA‐125 level was lower than its cutoff value (35 IU/mL)^22^ in two EOC samples (33 and 12.7 IU/mL) (Figure 3B) and higher in two benign samples (158 and 312 IU/mL) (Figure 3C). However, the Doppel level was consistently higher or lower than its cutoff value (2.67 ng/mL) in EOC (Figure 3B,C). These findings collectively validate our exploratory sera Doppel results and provide proof of concept of Doppel's usefulness as an OC biomarker over CA‐125 to distinguish and identify benign and high‐grade ovarian cancer patients.

### 3.3 Stratifying ovarian cancer based on Doppel level in patient's sera

Profiling of patients with high and low Doppel levels may be mapped to identify the significance of the Doppel axis in ovarian cancer progression. Based on the serum Doppel level, combining the EOC samples from our 1st and 2nd validation sets (*n* = 72), we proposed a Doppel index and classified them into Doppel‐high (Dpl^hi^) or Doppel‐low (Dpl^low^) groups. If the serum Doppel level is >6 ng/mL, we categorized them into Dpl^hi^. If the serum Doppel level is higher than the cutoff value (>2.67 ng/mL) but <6 ng/mL, we categorized them into Dpl^low^. A cutoff value of 6 ng/mL was selected to distinguish between the Dpl^hi^ and Dpl^low^ groups, as it falls between their respective mean values—18.11 ng/mL for Dpl^hi^ and 4.61 ng/mL for Dpl^low^. This threshold minimizes overlap between groups, as indicated by the SEM and SD values (Figure 3G). Control and benign samples, with mean Doppel levels of 0.73 and 1.21 ng/mL respectively, are also well below this threshold, reinforcing its discriminative power. To validate the 6 ng/mL cutoff, we performed a ROC curve analysis, which demonstrated high sensitivity and specificity for distinguishing between the groups (Figure S5). The optimal cutoff identified by Youden's index was 6.235 ng/mL, closely aligning with our selected threshold and further supporting its robustness. The observed Doppel levels at the boundaries of the two groups were 6.54 ng/mL (lowest in Dpl^hi^) and 5.93 ng/mL (highest in Dpl^low^), confirming that the 6 ng/mL threshold effectively separates them. Additionally, 95% confidence intervals (CIs) calculated using the formula CI = mean ± 1.96 × SEM support this demarcation. For the Dpl^low^ group, the 95% CI was ~4.24–4.98 ng/mL, placing 6 ng/mL above this range. For the Dpl^hi^ group, the 95% CI was ~10.98–25.24 ng/mL, placing 6 ng/mL well below this range. These results validate the use of 6 ng/mL as a meaningful and statistically sound cutoff. Furthermore, this stratification is consistent with tissue staining results, where Dpl^hi^ and Dpl^low^ groups showed significantly different H‐scores (Figures 3D,E and S2), further supporting the biological relevance of this threshold. Serum Doppel exhibited a significantly elevated level in both Dpl^hi^ (18.11 ng/mL; *n* = 37) and Dpl^low^ (4.61 ng/mL; *n* = 35) in comparison to control and benign groups (Figure 3F,G) and all found higher than LLOD. Moreover, in benign cases Doppel level was similar to control (1.2 ng/mL in benign and 0.74 ng/mL in control) with no significant differences (Figure 3F,G). The ROC curve achieved high accuracy for EOCs detection for both Dpl^low^ groups with an AUC of 0.99 and Dpl^hi^ groups with an AUC of 1.00 in comparison to control (Figure 3H,I). Similar results were obtained for both Dpl^low^ and Dpl^hi^ groups when the data were compared with benign groups (Figure 3I).

### 3.4 RNA sequencing of patient‐derived organoids from Doppel‐high versus low groups revealed induction of epithelial–mesenchymal transition in ovarian cancer cells

**FIGURE 4:**
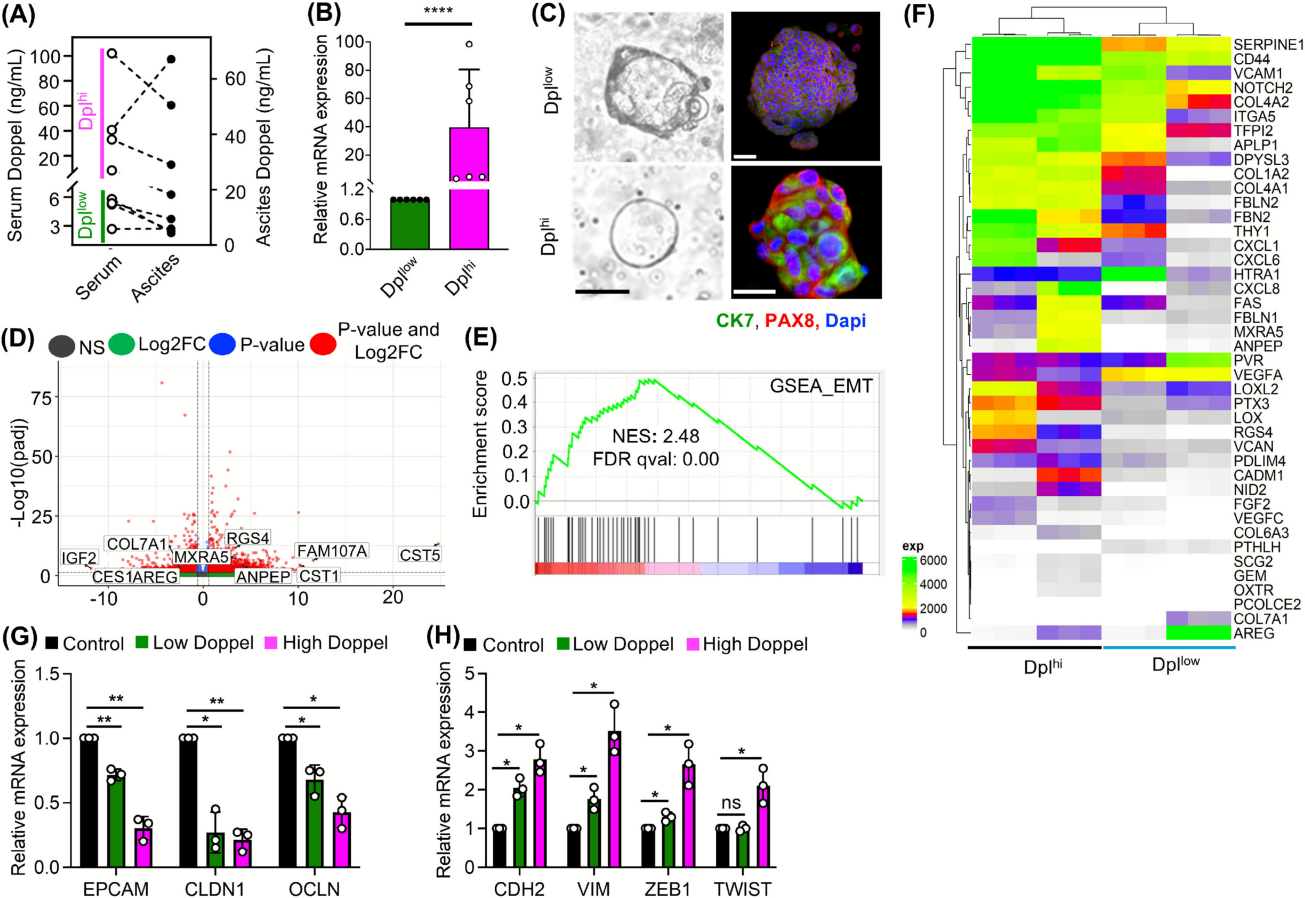
Doppel expression induced EMT in ascites‐derived organoids. (A) Pairwise comparison of serum Doppel with ascitic Doppel levels in ovarian cancer patients. (B) Doppel mRNA expression between Dpl^low^ and Dpl^hi^ in whole ascites‐derived cells. (C) Representative bright field (left) and immunofluorescence (right) images of organoids established from patient's ascites with Dpl^low^ and Dpl^hi^ levels. OC‐specific markers (CK7, PAX8) were used to confirm the establishment of organoids. Scale bars: 5 μm, 20 μm, and 200 μm. (D) Volcano plot of genes in Dpl^hi^ organoids compared with Dpl^low^ organoids; the y axis represents the negative Log 10 of *P* adjusted value; the *x* axis represents the Log2 fold changes between groups. (E) GSEA analysis showing the enrichment of EMT hallmark pathway in Dpl^hi^ organoids. (F) Heat map showing the EMT‐associated genes in Dpl^low^ versus Dpl^hi^ organoids. Normalized gene expression values are used. (G and H) Epithelial (G) and mesenchymal (H) specific marker gene expression respectively in SK‐OV‐3 cells treated with soluble Doppel. EMT, epithelial–mesenchymal transition; GSEA, gene set enrichment analysis; NES, normalized enrichment score; *P < .05, **P < .01.

The number of integrated and exploratory biomarkers in ovarian cancer detection is increasing, reflecting a potential clinical significance. Nevertheless, despite the anticipation surrounding their addition, many of these biomarkers frequently lack evidence of their involvement in the progression of ovarian cancer. The poor prognosis of ovarian cancer is associated with malignant ascites formation; fluid developed in OC patients' peritoneal cavity where cancer cells survive. Malignant ascites is common in ~75% of patients upon initial diagnosis and prevalent among patients who exhibit resistance to chemotherapy.^23^ This indicates that analyzing the molecular composition of malignant ascites in OC could offer valuable insights for clinical monitoring. At first, we checked Doppel level in eight patients' sera and ascites that were collected retrospectively (Table S2). All the patients were positive for Doppel detection based on our cutoff value (2.67 ng/mL) and the data revealed a strong correlation between sera and ascites Doppel level (Figure 4A), suggesting a role of Doppel in ascitic transformation. Four out of 8 patients had high Doppel expression in their sera and ascites that allowed us to categorize them into Dpl^hi^ and Dpl^low^ groups.

To understand the function of Doppel in malignant ascites, we chose four ascites samples, two each from the Dpl^hi^ and Dpl^low^ groups, with similar histology (serous and mucinous) types (Table S2). RT‐PCR analysis showed a significantly higher level of Doppel mRNA in the two Dpl^hi^ ascites than in the two Dpl^low^ ascites samples (Figure 4B), suggesting Doppel expression in the cellular fraction of ascites. Therefore, to uncover the phenotypical differences between the Dpl^hi^ and Dpl^low^ groups, we developed organoids from ascites‐derived cells following standard protocol.^17^, ^18^ Representative images of organoids show the morphological features of established organoids and expressions for OC markers: cytokeratin 7 (CK7) and paired box 8 (PAX8) (Figure 4C). We performed RNA sequencing using mRNA collected from ascitic organoids and identified a total of 1138 (549 up and 588 down) differentially expressed genes (DEGs) between Dpl^hi^ and Dpl^low^ groups based on fold change (−1 ≥ Log2FC ≥ 1), *P*adj ≤ .05, and count ≥10. Volcano plot shows all the genes including up and down in the Dpl^hi^ and Dpl^low^ groups (Figure 4D). To identify the significant biological processes of significant DEGs between the Dpl^hi^ and Dpl^low^ groups we performed hallmark gene‐set enrichment analysis (GSEA) and identified epithelial–mesenchymal transition (EMT) associated with the Doppel high group (Figure 4E). The heatmap depicting 42 genes related to EMT highlights the consistent disparities between the Dpl^hi^ and Dpl^low^ groups (Figure 4F). To confirm the involvement of Doppel in promoting EMT in the ascites TME, we treated SK‐OV‐3 cells, an ascites‐derived ovarian cancer cell line, with a Doppel‐high and Doppel‐low concentration for 24 hours. RT‐PCR analysis confirmed that Doppel significantly induces EMT by downregulating the epithelial‐specific markers (EPCAM, CLDN1, and OCLN) and upregulating the mesenchymal‐specific markers (CDH2, VIM, ZEB1, and TWIST) (Figure 4G,H). These findings confirmed that Doppel actively promotes EMT in the ovarian cancer ascites microenvironment.

### 3.5 Elevated Doppel level in the sera and ascites correlates with the dissemination of ovarian tumor cells into the circulation

**FIGURE 5:**
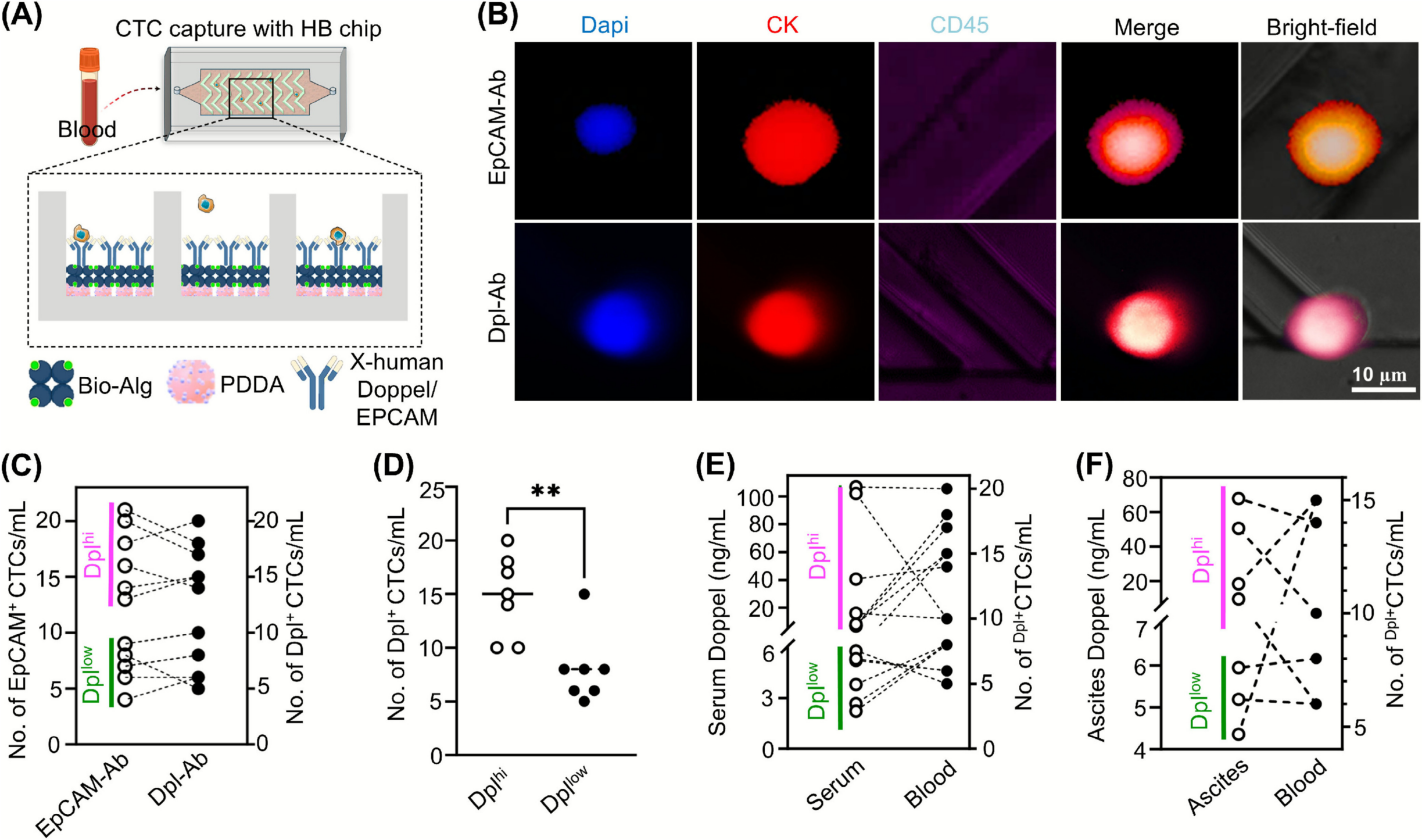
Serum and ascitic Doppel level correlate with circulating tumor cells in blood of EOC patients. (A) Schematic of microfluidic chip used to isolate EpCAM and Doppel expressed CTCs. (B) Representative immunofluorescence images of markers expression in isolated CTCs. Dapi^+^, CK^+^, CD45^−^ cells are considered CTCs. Upper panel: CTCs captured by EpCAM antibody‐coated chips and lower panel: CTCs captured by Doppel antibody‐coated chips. Scale bar: 10 μm. (C) Pairwise comparison of isolated ^EPCAM+^CTCs and ^Dpl+^CTCs in the patient's blood. (D) No. of ^Dpl+^CTCs isolated from 14 EOC patients' whole blood that are stratified into Dpl^hi^ and Dpl^low^ groups. (E) Pairwise comparison of serum Doppel level with whole blood ^Dpl+^CTCs. Correlation coefficient (*r* = .6025; *P* = .0125). (F) Pairwise comparison of ascites Doppel level with whole blood ^Dpl+^CTCs. Correlation coefficient (*r* = .1455; *P* = .3841). Ab, antibody; CK, cytokeratin; CTC, circulating tumor cells; Dapi, 4′,6‐diamidino‐2‐phenylindole; Dpl, Doppel; EpCAM, epithelial cell adhesion molecule. **P < .005.

If Doppel regulates EMT in ovarian cancer cells and shapes the ascitic TME, we hypothesize that Doppel may also control the dissemination of ovarian cancer cells into the circulation. To detect circulating tumor cells (CTCs) in the blood of Dpl^hi^ and Dpl^low^ patients, we devised an in‐house microfluidic chip model that was coated with Doppel antibody (Dpl‐Ab) (Figure 5A) and identified CTCs based on their expression of Dapi, EpCAM and cytokeratin, while noting the absence of CD45 expression in CTCs (Figure 5B). We detected and counted the Doppel‐expressing CTCs from fresh whole blood collected from 7 Dpl^hi^ and Dpl^low^ patients. As in the validation experiments, the anti‐EpCAM antibody was used as the capturing antibody and the fluorophore‐labeled cytokeratin as the target label. Overall, Dpl‐Ab coated chips showed similar efficiency of CTC capturing as with EpCAM‐ab coated chips (Figure 5C), which suggests CTCs exclusively express Doppel. Thus, we termed the Dpl‐Ab captured CTCs as ^Dpl+^CTC. ^Dpl+^CTC were significantly higher in the blood of Dpl^hi^ than Dpl^low^ patient groups (Figure 5D), strongly indicating the association of Doppel with OC dissemination into the circulation. Next, we performed a pairwise comparison of serum Doppel level with CTC counts in the blood. Despite some variability, the majority of Dpl^hi^ patients are associated with higher counts of CTCs in the blood (Figure 5E). The overall pairwise relationship between serum ascites and CTC counts in the blood showed significant correlation (*r* = .6025; *P* = .0125). We further performed the pairwise comparison between ascites Doppel level and CTC counts in the blood. Albeit the overall insignificant correlation (*r* = .1455; *P* = .3814), a correlation was established between ascites Doppel and the number of ^Dpl+^CTC in the blood (Figure 5F). These findings collectively established a novel link between serum/ascites Doppel levels and ovarian cancer dissemination into the circulation.

## 4 DISCUSSION

Taking into account the limitations of existing diagnostic modalities, in this study, we uncover for the first time that Doppel, a testis‐specific prion‐like protein, aberrantly expresses in OC tissues and its soluble form is detectable in patients' sera and ascites. Doppel can serve as a biomarker for OC, effectively distinguishing EOC patients, mostly in early stages of high‐grade serous cases from those with benign conditions and controls. Clustering the EOC patients into Dpl^hi^ and Dpl^low^ groups, we also identify how Doppel may influence ascitic TME and cancer cells to disseminate into the circulation. We demonstrate that Doppel influences the EMT pathway in ascitic TME, potentially resulting in a substantial increase in CTCs among OC patients.

Our study has two‐fold implications. First, the specificity and sensitivity of Doppel (AUC of 0.978 for Dpl vs. control) surpass that of CA‐125; CA‐125 was lower than its cutoff value (>35 IU/mL) in at least 20% of ovarian cancer cases in our cohorts as well as reported analyses.^24^ Further comparison between Doppel and CA‐125 for identifying benign conditions clearly indicates the superiority of Doppel over CA‐125. Importantly, we found an elevated Doppel level in a number of matched ascites of OC patients and a linear relationship between sera and ascitic Doppel level, indicating the involvement of Doppel in ascitic TME. Our data represent clinical significance, as ascites is found in over 90% of patients diagnosed with stage III/IV OC, and the correlation between ascites and disease progression is notable.^25^ In addition, ascites is a significant adverse predictor of outcome in recurrent disease.^23^ The observation that Doppel is not expressed in healthy ovaries, or any other reproductive organs of females suggests that this marker is a bona fide marker of neoplastic transformation. We identified serum Doppel as early as Stage I/II of EOCs and a significantly stepwise increase among HGSOCs, suggesting that Doppel expression would be important to detect the early stages of HGSOCs. Acquiring patient samples from multiple institutions also enables us to account for ethnic and geographical diversities. However, OC encompasses various invasive ovarian carcinomas originating from diverse tissues, including high‐grade serous, clear cell, endometrioid, and mucinous subtypes. HGSOC arises from the distal fallopian tube epithelium, but Doppel is not a specific marker for fallopian tube biology. Thus, how early the transformed epithelial cells express Doppel would be interesting to study. Understanding Doppel biology may deepen our insight into HGSOC's origin and early evolution.

Second, using the RNAseq approach with patient‐matched ascites and blood samples, we found that soluble Doppel level induces EMT ascitic TME and correlates with EOC aggressiveness. In the primary tumor tissues, as we have seen Doppel expression in tumor vasculatures as well as epithelial cells (Figure 1), this protein is known to regulate neo‐angiogenesis where the Doppel‐VEGFR2 axis plays the most crucial role.^14^ However, in malignant ascites, we found a causative role of Doppel with the EMT phenotype. The top two EMT‐related abundant genes in the Dpl^hi^ group, serpin family E member 1 (SERPINE1) and CD44, were reported to induce EMT in ovarian cancer.^26^, ^27^ Ascites has both cellular and acellular components, where acellular components can induce EMT.^28^ These findings suggest that soluble Doppel in ascites may prime cancer cells, thereby significantly contributing to the promotion of EMT. Further mechanistic investigation into the interaction between EMT genes and soluble Doppel could provide deeper insights into the mechanisms driving OC EMT in this specialized compartment.

Finally, we showed a link between CTC dissemination and Doppel expression. We found that the number of CTCs increased in the Dpl^hi^ OC group. This finding is interesting because we captured CTCs based on their ability to express Doppel. Doppel is known as a surface marker, and we observed Doppel expression both in the tumor endothelial and epithelial cells of primary tumors. In support of this study, we found that Doppel is exclusively detected in patient‐derived EOC organoids. Thus, the detection of ^Dpl+^CTC is not surprising in these transformed EOC cells. Also, previous studies demonstrate that CTCs with an EMT phenotype are common in OC patients.^29^ Since Doppel induces EMT and aids CTC dissemination in our patient‐matched cohorts, we posit that Doppel‐induced EMT may prime OC cells to disseminate into the circulation.

Although we provided proof‐of‐concept for Doppel as an OC biomarker, we have not directly assessed the association of Doppel expression with patient survival and mortality in our sample cohort. Future work should determine the effect of higher Doppel expression on patient outcomes using a large cohort of patients across the entire spectrum of the disease (e.g., benign, early, late stages as well as rare ovarian cancer subtypes). In addition, it will be interesting to check whether Doppel can be used to identify recurrence in patients with OC by using prospective samples, as the sensitivity of CA‐125 for recurrent EOCs varied widely (56%–94%).^30^ While we directly compared Doppel levels with those of CA‐125, the most widely used biomarker for OC in a clinical setting, additional comparisons with other emerging biomarkers such as HE4 or miRNA panels are warranted. Future studies should evaluate multi‐biomarker panels—incorporating Doppel, HE4, CA‐125, and validated miRNA signatures—in larger, prospective cohorts to firmly establish the clinical utility of Doppel. To examine Doppel's role in the ascitic TME, we utilized patient‐matched samples alongside advanced in vitro models and sequencing approaches. These strategies provided important insights into Doppel‐mediated EMT induction in OC. However, the limited number of ascites‐derived organoid RNA‐seq samples (*n* = 4) constrains both the statistical robustness and the generalizability of our findings. Expanding analyses to larger cohorts will be essential to better define the transcriptomic impact of Doppel on the OC ascitic TME.

We have not yet explicitly examined how Doppel induces EMT in the ascitic niche, but one plausible mechanism involves the TGF‐β pathway, a canonical EMT inducer enriched in OC ascites and known to synergize with angiogenic signaling.^31^ In OC, TGF‐β signaling activates pSMAD3 and the EMT transcription factor SNAIL, which represses epithelial markers such as E‐cadherin, thereby driving EMT and promoting a more invasive phenotype.^32^ We recently reported that overexpression of Doppel induces endothelial‐to‐mesenchymal transition, a process analogous to EMT, in pulmonary arterial hypertension through modulation of pSMAD3/SNAIL functions.^33^ Thus, Doppel may regulate TGF‐β signaling in OC ascites, thereby promoting EMT, and highlighting an exciting direction for future investigation. Beyond direct effects on EMT signaling, Doppel may act indirectly through its vascular effects, such as increasing permeability and reshaping the cytokine and growth factor milieu of ascites. Elevated ascitic Doppel could result from active secretion by tumor or tumor‐associated endothelial cells, as well as from passive leakage through hyperpermeable vasculature. Disentangling the relative contributions of these sources—and determining whether Doppel acts in synergy with TGF‐β or other EMT‐associated pathways—will require targeted in vitro and in vivo studies. Our paired correlation analysis further supports this model, showing a strong association between serum Doppel concentrations and ^Dpl+^CTC counts. This suggests a role for Doppel in facilitating OC dissemination into the circulation. Importantly, analysis of ^Dpl+^CTCs may provide insights into metastatic potential, while longitudinal monitoring of serum or ascitic Doppel and ^Dpl+^CTCs could hold significant promise for improving patient survival.

Collectively, our findings identified Doppel as a novel biomarker for OC diagnosis and highlight a mechanism of the Doppel axis in the OC ascitic TME. Our other major contribution is the detection of CTC based on Doppel expression, ^Dpl+^CTC. We showed two platforms, soluble Doppel in sera/ascites and ^Dpl+^CTC in whole blood, to detect subsets of EOCs that may be further developed for clinical validation. Also, ascites offer valuable insights into the primary tumor's progression to metastasis. Thus, targeting and studying the Doppel axis in ascites of EOCs may advance our understanding of key signaling pathways and identify a new therapeutic strategy.

## AUTHOR CONTRIBUTIONS

**Zulfikar Azam:** Writing – review and editing; writing – original draft; investigation; data curation; software; formal analysis; validation; visualization; methodology. **Xiaojun Zhang:** Investigation; formal analysis. **Riajul Wahab:** Investigation; formal analysis. **Md Mahedi Hasan:** Methodology; investigation; formal analysis; visualization; writing – original draft; writing – review and editing. **Shivaniben Dhadhal:** Investigation; formal analysis. **Bowon Kang:** Investigation; visualization. **Md Mynul Hassan:** Investigation; formal analysis; writing – original draft. **Mazharul Karim:** Investigation. **Jeong Uk Choi:** Investigation; resources. **Muhit Rana:** Investigation; resources. **Jian‐Ying Zhang:** Resources. **Sourav Roy:** Formal analysis. **Youngro Byun:** Resources. **In‐San Kim:** Resources. **Jae Yun Song:** Resources. **Eugene P. Toy:** Resources. **Sireesha Y. Reddy:** Resources. **Farzana Alam:** Writing – original draft; investigation; formal analysis; data curation. **Taslim A. Al‐Hilal:** Conceptualization; investigation; funding acquisition; writing – original draft; methodology; validation; visualization; writing – review and editing; software; formal analysis; project administration; data curation; supervision; resources.

## CONFLICT OF INTEREST STATEMENT

The authors declare no conflict of interest.

## ETHICS STATEMENT

Written informed consent was obtained from all participants prior to their inclusion in the study. This study was conducted in accordance with the ethical principles outlined in the World Medical Association Declaration of Helsinki and was approved by the ethics committee of Texas Tech University Health Sciences Center El Paso (IRB no.: E21144). All data were anonymized, and strict measures were implemented to ensure confidentiality.

## Supporting information


**Table S1.** Demographic information of recruited participants.
**Table S2.** Bio‐banked ascites and ascites‐derived organoids (AsO) with clinical pathology.
**Table S3.** RNA sequencing coverage and quality statistics.
**Figure S1.** Validation of Doppel antibodies.
**Figure S2.** Representative IHC images of all Doppel stained OC tissue samples with 2B6 antibody.
**Figure S3.** Prognostic impact of Doppel expression in public ovarian cancer datasets.
**Figure S4.** Serum Doppel level is elevated in training set of ovarian cancer patients.
**Figure S5.** ROC curve to stratify Doppel index.

## Data Availability

The RNA‐seq raw FASTQ files generated in this study are available in Zenodo (https://doi.org/10.5281/zenodo.16786227). Other data that support the findings of this study are available from the corresponding author upon request.
