# Supplementary material for: Doppel as an early‐stage biomarker promoting EMT and dissemination in ovarian cancers
Source: Int J Cancer. 2025 Nov 29;158(6):1755–68. doi: 10.1002/ijc.70268 (PMC12811216; doi:10.1002/ijc.70268)
Supplement: Supplementary file 1 — Table S1. Demographic information of recruited participants. Table S2. Bio‐banked ascites and ascites‐derived organoids (AsO) with clinical pathology. Table S3. RNA sequencing coverage and quality statistics. Figure S1. Validation of Doppel antibodies. Figure S2. Representative IHC images of all Doppel stained OC tissue samples with 2B6 antibody. Figure S3. Prognostic impact of Doppel expression in public ovarian cancer datasets. Figure S4. Serum Doppel level is elevated in training set of ovarian cancer patients. Figure S5. ROC curve to stratify Doppel index. [file IJC-158-1755-s001.pdf]

Supplementary Material for

Doppel as an early-stage biomarker promoting EMT and dissemination in ovarian cancers

Zulfikar Azam, Xiaojun Zhang, Riajul Wahab, Md Mahedi Hasan, Shivaniben Dhadhal, Bowon Kang, Md Mynul Hassan, Mazharul Karim, Jeong Uk Choi, Muhit Rana, Jian-Ying Zhang, Sourav Roy, Youngro Byun, In-San Kim, Jae Yun Song, Eugene P. Toy, Sireesha Y. Reddy, Farzana Alam, Taslim A. Al-Hilal

Table of contents:

Supplementary Tables S1 – S3

Supplementary Figures S1 – S5

Table S1: Demographic information of recruited participants

Demographics	Control	Cases
**N**	17	22
**Age, year, mean (SD)**	50.41 (12)	60.09 (14.4)
**≥55**	12	10
**<55**	5	12
**Height, inch, mean (SD)**	63.06 (2.21)	62.87 (3.53)
**Weight, lb, mean (SD)**	179.38 (46.39)	150.83 (23.51)
**BMI, mean (SD)**	31.44 (6.2)	26.46 (4.01)
**Ethnicity**		
Hispanic	16(94.12%)	18 (81.82%)
Non-Hispanic	4 (18.18%)	4 (18.18%)
**Race**		
White	15(88.26%)	19(86.36%)
Others	1(5.88%)	2(9.09%)
Unknown		1(4.55%)
Asian	1(5.88%)	
**Physical Activity**		
Yes	12(70.59%)	9(40.91%)
No	4(23.53%)	13(59.09%)
Unknown	1(5.88%)	
**Family history of Ovarian cancer**	0 (0.00%)	1 (4.55%)
**Family history of other cancers**	9 (40.41%)	6 (42.86%)
**FIGO Stage**	N/A	Benign (7), I (2), II (1), III (10), Unknown (1)

Table S2: Bio-banked ascites and Ascites-derived organoids (AsO) with clinical pathology

					Ascitic fluid	
**Pt. No.**	**Ethnicity**	**Race**	**EOC Type (FIGO Stage)**	**Doppel level**	**AsO established**	**RNA-Seq of AsO**
#27	Hispanic	White	Mucinous Adenocarcinoma (IIB)	Low	Yes	Yes
#24	Hispanic	Mixed race	Serous Grade 3 (IIIC)	High	Yes	Yes
#31	Hispanic	White	Carcinosarcoma (IIIC)	High	N/A	
#40	Hispanic	White	Low grade serous cancer (III A2)	High	N/A	
#44	Hispanic	White	Mucinous Adenocarcinoma (IIA)	High	Yes	Yes
#46	Hispanic	White	Serous Grade 3 (IIIA)		No Ascites	
#47	Hispanic	While	Serous Grade 3 (IV)		No Ascites	
#48	Hispanic	White	Serous Grade 3 (IIIC)	Low	Yes	Yes

Table S3: RNA sequencing coverage and quality statistics

Sample ID	Total number of sequenced reads	Total number of uniquely mapped reads (GRCh38)	RNA integrity number (RIN)	Ratio of all reads aligned to rRNA regions to total uniquely mapped reads (rRNA rate)	Ratio of exon-mapped reads to total uniquely mapped reads (Expression Profile Efficiency)	Total number of detected transcripts with reads ≥1
Pt24AsOr1	55025438	51203094	8.4	4.59%	94.07%	25871
Pt24AsOr2	48832028	45663877	8.3	4.97%	93.47%	25705
Pt24AsOr3	45573556	42619387	8.6	4.85%	93.49%	25449
Pt27AsOr1	38710408	35470901	7.7	5.65%	90.06%	25570
Pt27AsOr2	35876020	32935450	7.7	5.56%	90.47%	25233
Pt27AsOr3	35681568	32761839	7.8	5.93%	90.90%	25019
Pt44AsOr1	44377914	41593056	9.8	3.87%	91.43%	25304
Pt44AsOr2	59738272	55991796	9.8	3.67%	91.81%	26077
Pt44AsOr3	59774514	55863931	9.8	3.34%	91.65%	26117
Pt48AsOr1	39664142	36540478	9.4	3.95%	89.44%	24653
Pt48AsOr2	40534498	37475581	9.9	3.92%	89.40%	24839
Pt48AsOr3	40727268	37571723	9.9	3.88%	89.37%	24627

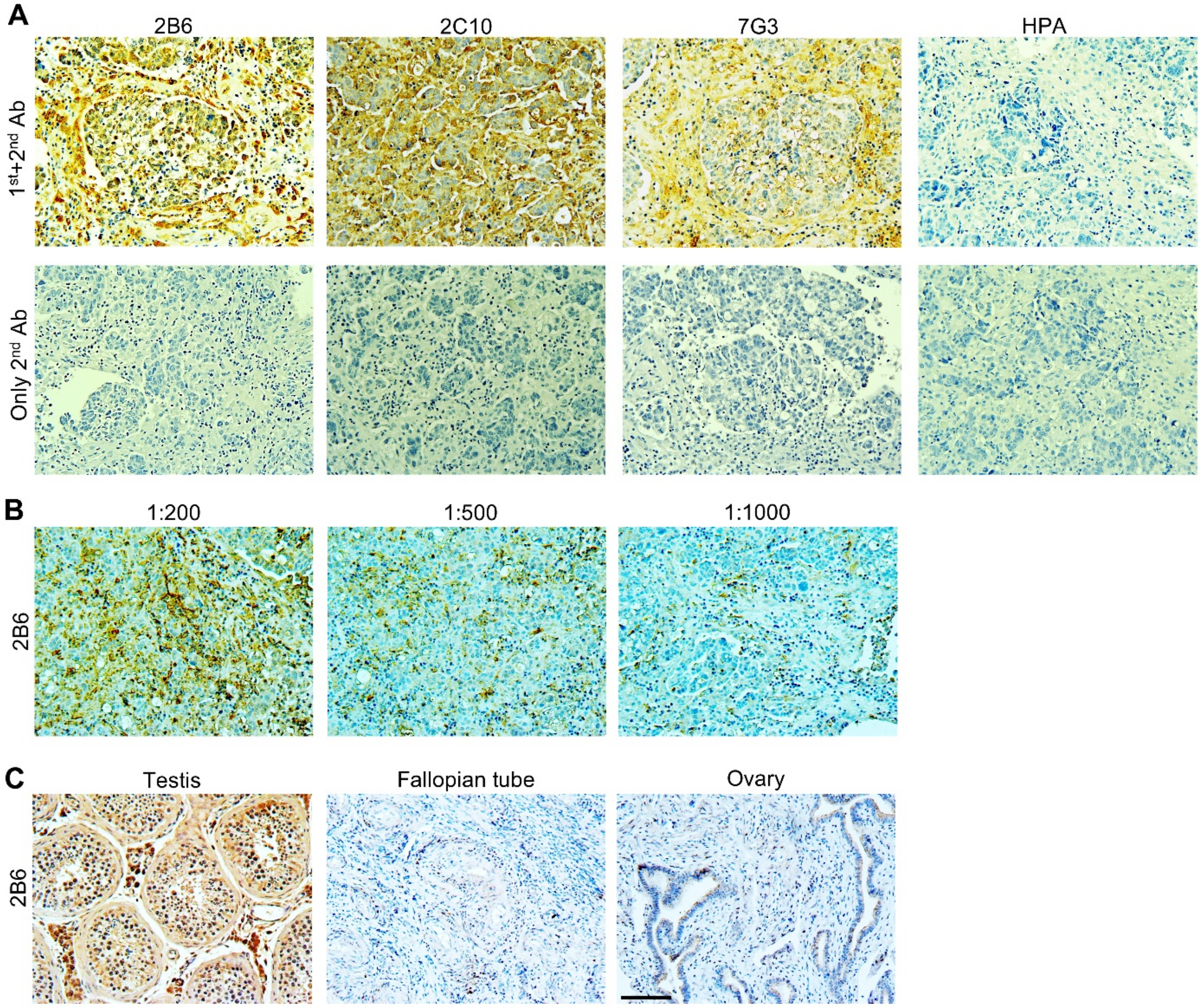

Figure S1: Validation of Doppel antibodies. (**A)** IHC validation of three custom made antibodies (2B6, 2C10, and 7G3) and one-commercially available antibody (HPA) to stain Doppel in OC tissues. (**B)** Validation of 2B6 antibody dilution. (**C)** Representative IHC images of 2B6 antibody staining of Doppel in positive control testis and normal fallopian and ovary tissue samples. Ovary image is reused in Fig 1B as Ovary. Scale bar- 50 µm.

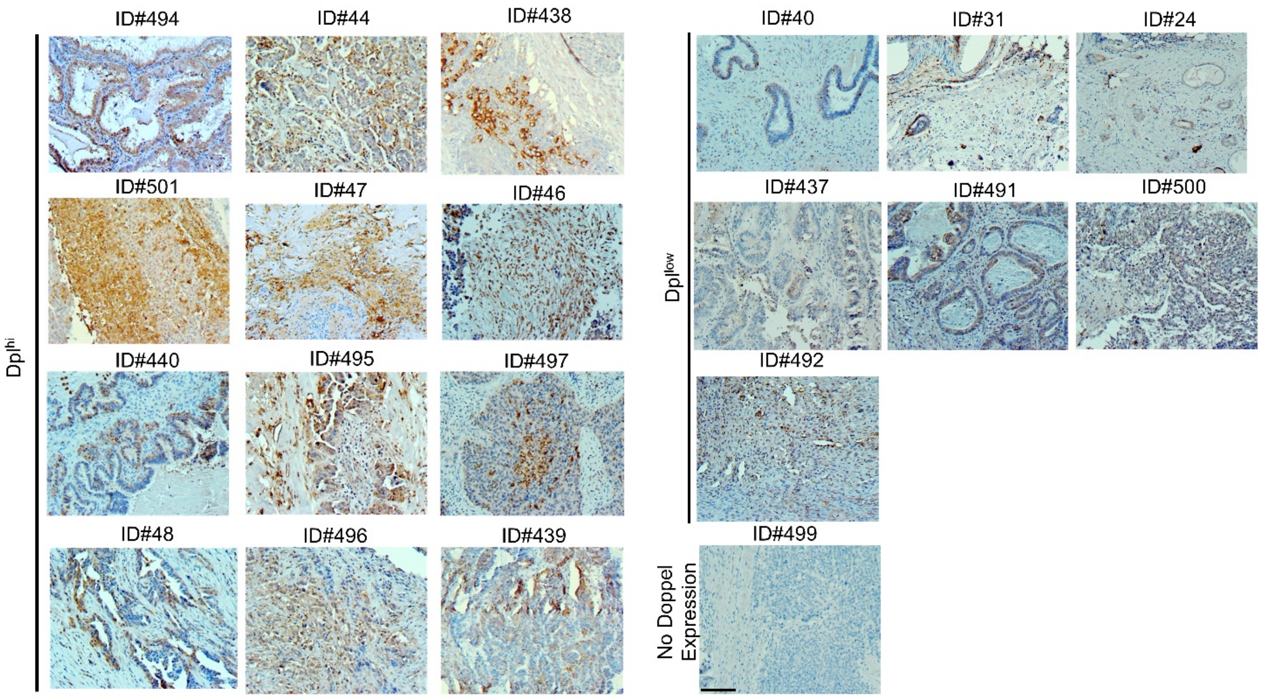

Figure S2: Representative IHC images of all Doppel stained OC tissue samples with 2B6 antibody. ID#495 image is reused in Fig 1B as EOC#1. ID#494 and ID#491 images are reused in Fig 3D as Dpl^{hi} and Dpl^{low} respectively. Scale bar- 50 µm**.**

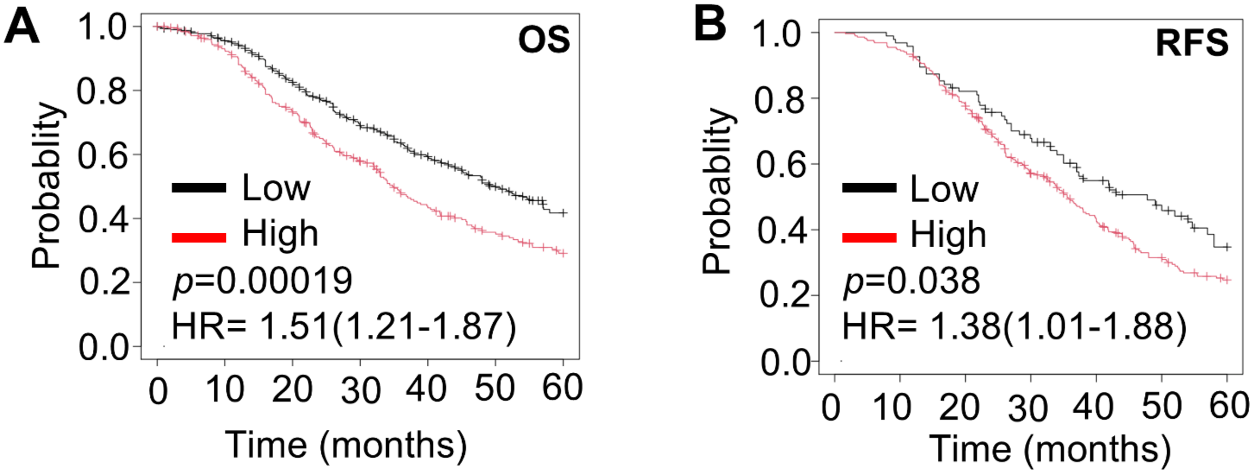

Figure S3: Prognostic impact of Doppel expression in public ovarian cancer datasets. (A) Kaplan-Meier overall survival analysis of public human serous ovarian cancer patients in the indicated groups with different Doppel levels. **(B)** Kaplan-Meier regression free survival analysis of public human serous ovarian cancer patients in the indicated groups with different Doppel levels.

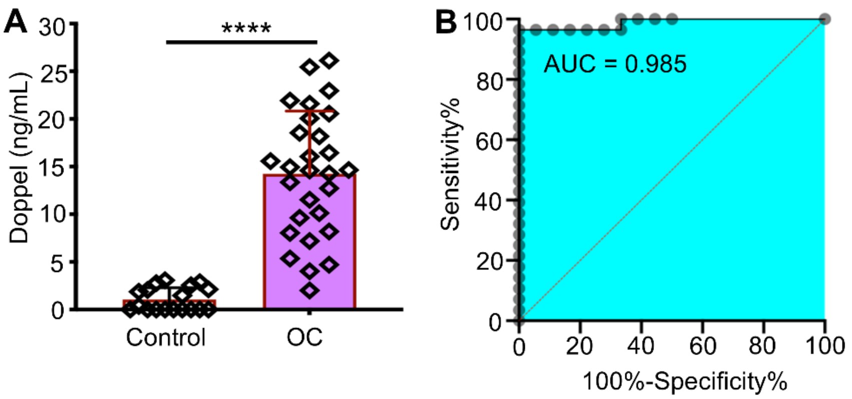

C

	Control	OC
**Cases No.**	18	28
**Mean (ng/mL)**	1.06	14.24
**SD**	1.23	6.58
**Positive**	0/18	26/28

Figure S4: Serum Doppel level is elevated in training set of ovarian cancer patients. (A) Measurement of serum Doppel of ovarian cancer and control subjects by ELISA. Error bar represents mean +SD. **(B)** The ROC curve of Doppel between control and ovarian cancer serum samples. **C)** Serum Doppel level comparison chart between control and ovarian cancer subjects.
****= <0.0001.

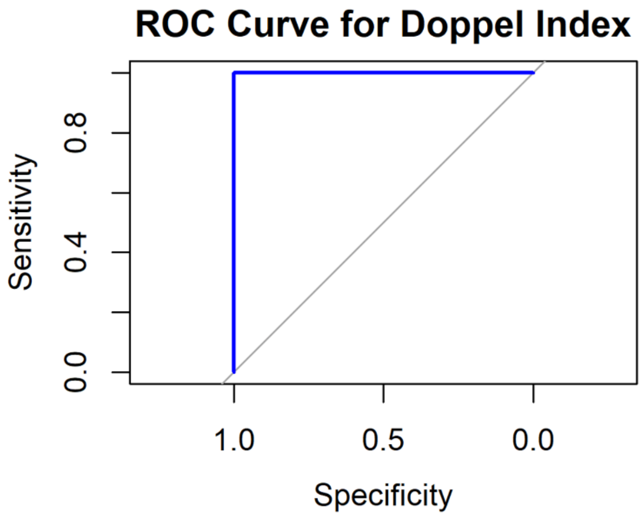

Figure S5: ROC curve to stratify Doppel index.
